# Systemic lupus erythematosus presenting with pneumoperitoneum without evidence of pneumatosis cystoides intestinalis—when not to operate—a case report

**DOI:** 10.1093/jscr/rjae182

**Published:** 2024-03-27

**Authors:** Wafa Iftekhar, Hafsa Shaikh, Abdul R Alvi

**Affiliations:** Department of General Surgery, Aga Khan University Hospital, Stadium Road, P.O. Box 3500, Karachi 74800, Pakistan; Department of General Surgery, Aga Khan University Hospital, Stadium Road, P.O. Box 3500, Karachi 74800, Pakistan; Department of General Surgery, Aga Khan University Hospital, Stadium Road, P.O. Box 3500, Karachi 74800, Pakistan

**Keywords:** case report, systemic lupus erythematosus, spontaneous pneumoperitoneum, pneumatosis cystoides intestinalis

## Abstract

Pneumoperitoneum in patients with systemic lupus erythematosus is commonly recognized as a surgical emergency that requires exploration. However, it may not be associated with bowel perforation and may be a benign disease manifestation. We present a case of a young patient who developed spontaneous pneumoperitoneum after pulse steroid therapy for lupus enteritis and was successfully managed conservatively. Patients with connective tissue disease may present with pneumoperitoneum, with or without pneumatosis cystoides intestinalis. Therefore, a detailed clinical history, thorough clinical examination, and laboratory parameters should be evaluated before proceeding with surgical intervention. A conservative approach may be attempted in patients with spontaneous pneumoperitoneum, and surgery should only be considered if clinical deterioration occurs.

## Introduction

The pneumoperitoneum is the presence of free in the peritoneal cavity. In 1915, Hugo Popper first described using radiographic methods to detect pneumoperitoneum. Diagnosing pneumoperitoneum requires a thorough history, physical examination, and imaging tests like an erect chest X-ray and computed tomography (CT). Multidetector CT is the preferred diagnostic tool for suspected perforation, thanks to its high sensitivity in detecting extraluminal gas and ability to accurately locate the perforation site, with an accuracy ranging from 82% to 90% [1]. However, using water-soluble iodinated oral contrast is still a matter of debate. Oral contrast can significantly delay management, obscure radiopaque foreign bodies, and may be poorly tolerated. Though, if present, oral contrast leakage is a highly specific sign for localizing the perforation site, it carries low sensitivity ranging from 19% to 42% [1].

Pneumoperitoneum commonly occurs as a result of perforation in the gastrointestinal tract with inflammatory conditions being the most common cause and the gastroduodenal area being the most common site of perforation [2]. This accounts for 85%–90% of all cases of pneumoperitoneum and commonly requires surgical intervention. However, 5%–15% of cases present without evidence of visceral perforation [3] and are known to have spontaneous or nonsurgical pneumoperitoneum.

Spontaneous pneumoperitoneum can occur as a result of intrathoracic, intraabdominal, and gynecological causes, chest trauma, cardiopulmonary resuscitation, and ventilated patients [4]. Regarding abdominal causes, it is most commonly associated with pneumatosis cystoides intestinalis [4].

Pneumatosis cystoides intestinalis, also known as cystic lymphomatosis or enteromesenteric emphysema, was first described by DuVernoi during a cadaver dissection in 1730. The development of pneumatosis cystoides intestinalis has been associated with various medical conditions such as idiopathic development, scleroderma, bone marrow transplant, AIDS, diverticular disease, small bowel resection, intestinal pseudo-obstruction, dermatomyositis, nontropical sprue, jejunal-ileal bypass, gastric outlet obstruction, sclerotherapy, heart transplantation, and respiratory diseases [5, 6]. Gas-filled cysts can be found in any part of the gastrointestinal tract but are most common in the terminal ileum [5]. They can also be found in the stomach and colon. Constant filling, rupturing, sealing, and refilling of cysts lead to spontaneous pneumoperitoneum. The condition typically resolves on its own but may recur. Four hypotheses have been proposed for this process: bowel necrosis, gas from the gastrointestinal tract, gas-producing bacteria, and gas from alveoli [5].

Other reasons for spontaneous pneumoperitoneum include the placement of percutaneous endoscopic gastrostomy and biliary stents. Rare abdominal causes include emphysematous cholecystitis, spontaneous bacterial peritonitis, ruptured liver abscess, and perforated pyometra in women [6]. Finally, there are patients for whom there are no identifiable risk factors and causes for pneumoperitoneum and who are categorized as having idiopathic spontaneous pneumoperitoneum [7, 8].

Identification of spontaneous pneumoperitoneum and awareness of the causes allow conservative management of selected patients without any indications of surgery [4]. This may prevent unnecessary surgical intervention and additional morbidity and mortality associated with negative laparotomies. Herein, we report a case of a young female with systemic lupus erythematosus who was reported to have spontaneous pneumoperitoneum without any evidence of pneumatosis cystoides intestinalis on imaging, which was managed conservatively.

## Case report

A young female in her early 20’s, diagnosed with systemic lupus erythematosus 4 years back was referred to the emergency department of our academic institution. Her past medical history included multiple admissions for SLE enteritis with the last admission being five years back. Her home medications included Azathioprine 50 mg twice a day and prednisolone 5 mg twice a day.

She now presented with progressively worsening abdominal pain with nausea and vomiting for 10 days. She had been hospitalized and was treated by rheumatologist for SLE enteritis with pulse steroid therapy using methylprednisolone 1 g thrice a day. However, her symptoms worsened, and she underwent a CT scan abdomen with contrast, which showed gross pneumoperitoneum. She was referred to a tertiary care center for multidisciplinary management and surgical intervention.

At the time of the presentation, she appeared pale and dehydrated. She was conscious, oriented, and afebrile. Her blood pressure was 110/70 mm Hg, heart rate was 140 beats/min, and peripheral oxygen saturation was 96% on room air. Chest examination was normal. The abdomen was distended, soft, and non-tender with no clinical evidence of peritonitis. Her neurological assessment was normal.

Laboratory assessment showed hemoglobin 12.6; leukocytes 8.9 with neutrophils shift to 92.7%; thrombocytopenia (120 000 per microliter of blood); CRP 195.43 mg/dl; creatinine 0.5 mg/dL; urea 7 mg/dL; Lactate 1.4 mmol/L and base deficit −0.8 mEq/L.

**Figure 1:**
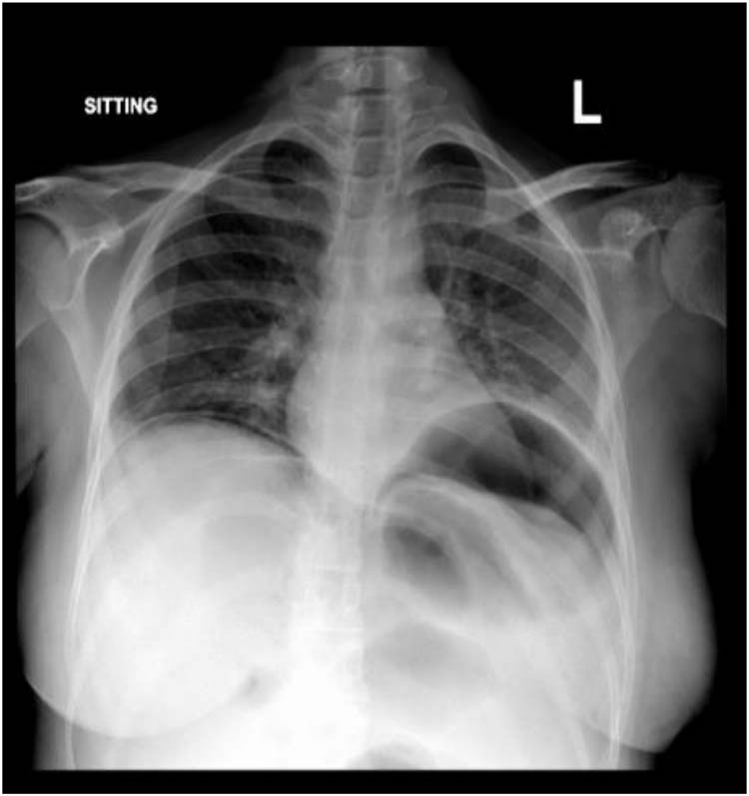
Chest X-ray.

Subdiaphragmatic free air was detected on the posteroanterior chest radiograph, without air insufflation by nasogastric tube (Fig. 1).

**Figure 2:**
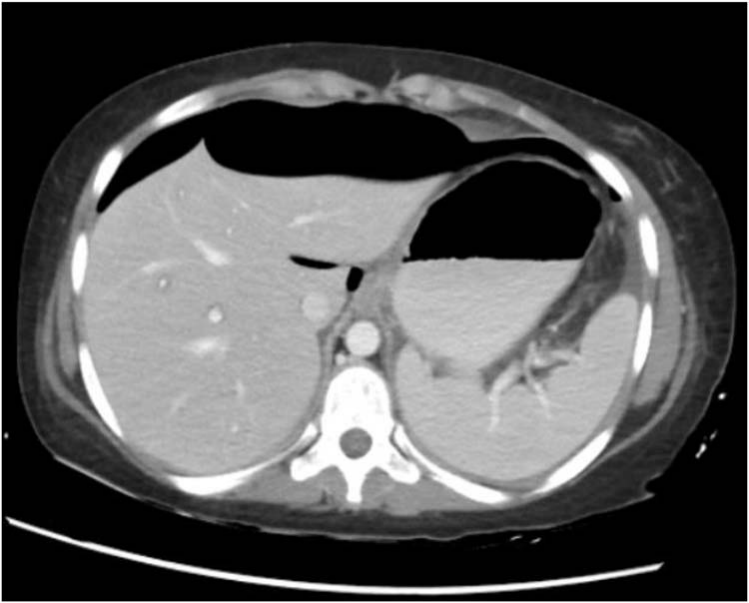
CT abdomen with oral and IV contrast.

CT abdomen with oral and intravenous contrast was done, which revealed gross pneumoperitoneum, significantly dilated stomach with dilated multiple small bowels loops up to the distal ileum with no evidence of contrast extravasation. Moderate ascites with mesenteric congestion were seen (Fig. 2).

She was resuscitated with intravenous fluids after which her heart rate improved to normal. A multidisciplinary approach was taken and rheumatologist was consulted for opinion. After discussion and considering the patient’s clinical condition, a conservative management approach was planned with closed observation in the special care unit under the care of a rheumatologist. She received IV Meropenem; her oral intake was restricted and was started on total parenteral nutrition. The next day, she was started on high dose methylprednisolone 60 mg 8 hourly. Her abdominal symptoms and signs improved over the next 5 days. She had her bowel movements on the next day of admission and tolerated the enteral diet and was discharged on the 5th day of admission on oral prednisolone 15 mg thrice a day and IV Meropenem. She was planned for monthly pulse cyclophosphamide for 6 months once recovered.

She was seen in the clinic 1 week later with a repeat CT scan. She had complete resolution of her symptoms and the imaging showed interval resolution of previously noted gross pneumoperitoneum.

## Discussion

Systemic lupus erythematosus, an autoimmune inflammatory disease, is commonly associated with abdominal symptoms. Lupus enteritis is one of the most common causes of abdominal pain in SLE patients [9]. Lupus enteritis symptoms vary from mild abdominal pain to severe conditions like necrosis and perforation, which present as gastrointestinal bleeding or peritonitis. It is worth noting that typical signs of bowel perforation may be absent in lupus enteritis patients.

The other complication that can present with pneumoperitoneum in patients with systemic lupus erythematosus is pneumatosis cystoides intestinalis which being rare has been identified in only 17 reported cases [10–12]. Adachi *et al*. [13] reported in a retrospective article that pneumatosis cystoides intestinalis were observed in 24.7% of cases with pneumoperitoneum, being the second most common cause of pneumoperitoneum. Pneumatosis may lead to spontaneous pneumoperitoneum because of the rupture of subserosal cysts.

However, in our case above, we report a spontaneous pneumoperitoneum thought to be because of systemic erythematosus without any evidence of pneumatosis cystoides intestinalis and no evidence of contrast extravasation on imaging in the emergency department. It is possible that the pneumatosis cystoides resolved or went unnoticed during the CT scan. Some patients have experienced recurrent pneumoperitoneum with pneumatosis cystoides [14]. Additionally, pneumatosis cystoides and/or pneumoperitoneum can disappear quickly. These observations suggest that extraluminal free air may be detected in pneumoperitoneum cases caused by pneumatosis when CT examination is delayed [13]. Therefore, pneumatosis cystoides may be one of the reasons for extraluminal free air in idiopathic pneumoperitoneum cases [13].

Mizoguchi *et al*. [10] have reported a case of pneumatosis cystoides after high-dose prednisolone therapy for lupus enteritis and have suggested lupus enteritis and high-dose prednisolone treatment as risk factors for the development of pneumatosis cystoides intestinalis. Likewise, our patient was recently treated with pulse therapy for lupus enteritis and pneumatosis intestinalis though not visible on initial imaging might be the cause of spontaneous pneumoperitoneum.

The presence of pneumoperitoneum is generally considered an ominous sign of a surgical emergency. However, not all pneumoperitoneum require surgical intervention. It has been observed that in some cases, surgeons may face challenges when it comes to accurately diagnosing and determining the appropriate treatment for patients who have spontaneous pneumoperitoneum and have performed exploratory laparotomies [15, 16].

The patient’s history, clinical examination, and laboratory data should be correlated with the CT findings [17]. Patients with mild symptoms, benign findings on clinical examination, and no laboratory and imaging evidence of sepsis, bowel ischemia, or perforation can be diagnosed to have non-surgical or spontaneous pneumoperitoneum and can be managed conservatively [6, 16, 18]. Conservative treatment can be started with intravenous antibiotic therapy, nasogastric decompression, and bowel rest. Surgical treatment is reserved for cases with acute abdomen, suspected complications, and failed conservative treatment [19]. When faced with uncertainty, laparoscopic exploration can be a viable and effective alternative to laparotomy [7].

Early recognition and watchful monitoring can prevent unnecessary investigations and surgical intervention. Since our patient had a history of systemic lupus erythematous, was recently treated for lupus enteritis with high dose immunosuppressant, was clinically stable with no evidence of peritonitis, and demonstrated no obvious source of visceral perforation this led us to believe that it was a benign spontaneous pneumoperitoneum and conservative approach was chosen for her.
